# Influenza, swine flu, sperm quality and infertility: A story

**DOI:** 10.4103/0974-1208.69339

**Published:** 2010

**Authors:** Viroj Wiwanitkit

**Affiliations:** Department of Laboratory Medicine, Faculty of Medicine, Chulalongkorn University, Bangkok - 10330, Thailand

Sir,

Infection is accepted as a possible underlying cause of male infertility. Some infections are confirmed for the negative effect on sperm quality, for example, genital tract infection.[1] In addition to bacteria, other pathogens can also affect semen quality and result in infertility as a consequence.[2] The roles of parasites and fungi in induction of infertility are confirmed.[2] The most well-known virus-induced infection leading to infertility is mumps. Indeed, mumps result in orchitis. López Pacios *et al*. studied on eight cases with mumps-induced orchitis and concluded that “long-term follow-up is recommended for all patients with abnormal semen analysis, particularly those with bilateral testicular involvement, since they may develop oligoasthenospermia several years after the infection or improve with item”.[3] However, the role of other viral infections is still limitedly reported. In this specific article, the author discusses the effect of influenza infection on sperm quality and further extrapolates the discussion on the present emerging swine flu.

The influenza is a respiratory virus infection. It mainly affects the respiratory tract. However, there are some reports on the effect of influenza on sperm quality. As a febrile illness, influenza might affect the semen quality. Sergerie *et al*. reported that a febrile episode could have marked effects on semen parameters and sperm DNA integrity and this might be related to future infertility.[4] Evenson *et al*. studied the characteristics of human sperm chromatin structure following an episode of influenza and high fever and found that influenza could have latent effects on sperm chromatin structure and might result in transient release of abnormal sperm.[5] Indeed, there are some reports on animal models confirming that influenza can impair sperm quality and result in infertility. Devi *et al*. studied in a mice model and reported that human influenza virus could induce chromosome aberration of spermatozoa.[6] Thadani and Polasa also confirmed that inoculation of formaldehyde-inactivated A2 Hong Kong influenza virus resulted in the higher percentages of chromosome aberrations in mouse spermatogonia than were seen in controls.[7,8] Sharma and Polasa also observed and reported similar findings.[9] These are considered as the effect of the pyrogenic and hemagglutinating properties of influenza viruses that might be seen in case of human beings.[10]

Hence, it can be concluded that there are strong evidences confirming the effect of influenza infection on sperm quality. The query if influenza can induce future infertility is interesting. There is a lack of evidence on this topic. Indeed, there are some reports confirming that influenza might lead to infertility in animals but not in human beings.[11] However, if there is an actual latent effect of influenza on sperm as proposed by Evenson *et al*.[5] this might lead to infertility. As a conclusion, further researches are still needed to explore the correlation between influenza and infertility. Nevertheless, it should be noted that the influenza vaccination is recommended for the infertile subjects.[12]

Emerging swine flu is a kind of variant of classical H1N1 influenza virus infection and hence it is doubtless that swine flu can affect the sperm quality. At least, the acute febrile episode can affect the sperm. However, there exist no official reports on this aspect as well as the correlation between swine flu and infertility. Nevertheless, the presentation and effect of swine flu in infertile case has never been well explored.
